# Crystal structure and Hirshfeld surface analysis of the new cyclo­diphosphazane [EtNP(S)NMe_2_]_2_

**DOI:** 10.1107/S2056989017005187

**Published:** 2017-04-11

**Authors:** Chokri Issaoui, Hammouda Chebbi, Khaled Alouani, Abderrahmen Guesmi

**Affiliations:** a University of Tunis El Manar, Faculty of Sciences of Tunis, Laboratory of Materials, Crystal Chemistry and Applied Thermodynamics, 2092 El Manar II,Tunis, Tunisia; b Preparatory Institute for Engineering Studies of Tunis, Street Jawaher Lel Nehru, 1089 Montfleury, Tunis, Tunisia; c University of Tunis El Manar, Faculty of Sciences of Tunis, Laboratory of Electrochemistry, 2092 El Manar II,Tunis, Tunisia

**Keywords:** crystal structure, cyclo­diphosphazane, [EtNP(S)NMe_2_]_2_, Hirshfeld surface analysis, fingerprint plots

## Abstract

The cyclic compound 2,4-bis(dimethylamino)-1,3-diethylcyclodiphosphazane-2,4-dithione [systematic name: 2,4-bis(dimethylamino)-1,3-diethyl-1,3,2λ^5^,4λ^5^-diazadiphosphetidine-2,4-dithione], C_8_H_22_N_4_P_2_S_2_ or [EtNP(S)NMe_2_]_2_, is member of a class of mol­ecules that may be used, by virtue of their complexation properties, for the extraction of metals. This compound was characterized in solution by (^1^H and ^31^P) NMR, and in the solid state by energy-dispersive X-ray spectroscopy (EDX) and by X-ray crystallography. In the crystal, the mol­ecule sits on an inversion centre such that the P and N atoms form a centrosymmetric cyclic P_2_N_2_ arrangement. The crystal packing is dominated by van der Waals inter­actions. The prevalence of these inter­actions is illustrated by an analysis of the three-dimensional Hirshfeld surface (HS) and by two-dimensional fingerprint plots (FP). The relative contribution of different inter­actions to the HS indicates that the H⋯H contacts account for 74.3% of the total HS area.

## Abstract 2

A new cyclo­diphosphazane, [EtNP(S)NMe_2_]_2_, was synthesized and characterized by NMR and EDX spectroscopy and single-crystal XRD. The stability of the structure is ensured only by van der Waals inter­actions and the their prevalence is confirmed by an analysis of the three-dimensional Hirshfeld surface (HS) and two-dimensional fingerprint plots (FP).

## Chemical context   

In the study of organo­phospho­rus compounds, one of the aims is to prepare new complexing agents. Indeed, the literature shows many studies of the bidentate organo­phospho­rus ligands HN[P(*E*)R_2_]_2_ (*E*: O, S, Se; Balazs *et al.* 1999 ▸; Silvestru *et al.* 2000 ▸; Ghesner *et al.* 2005 ▸; Cristurean *et al.* 2008 ▸) and *R*N[P(*E*)R_2_]_2_ (Benabicha *et al.* 1986 ▸; Ladeveze *et al.* 1986 ▸; Alouani *et al.* 2002 ▸, 2007 ▸; Peulecke *et al.* 2009 ▸), *etc*. All of these ligands may act as chelating agents containing both hard (N) and soft (P) elements. In addition, the flexibility of the (EPNPE) system provides a ready means of altering, and thereby possibly improving, their complexing properties. Several complexes based on these ligands have been reported, such as those described by Bennis & Alouani (2012 ▸) and by Mejri *et al.* (2016 ▸).
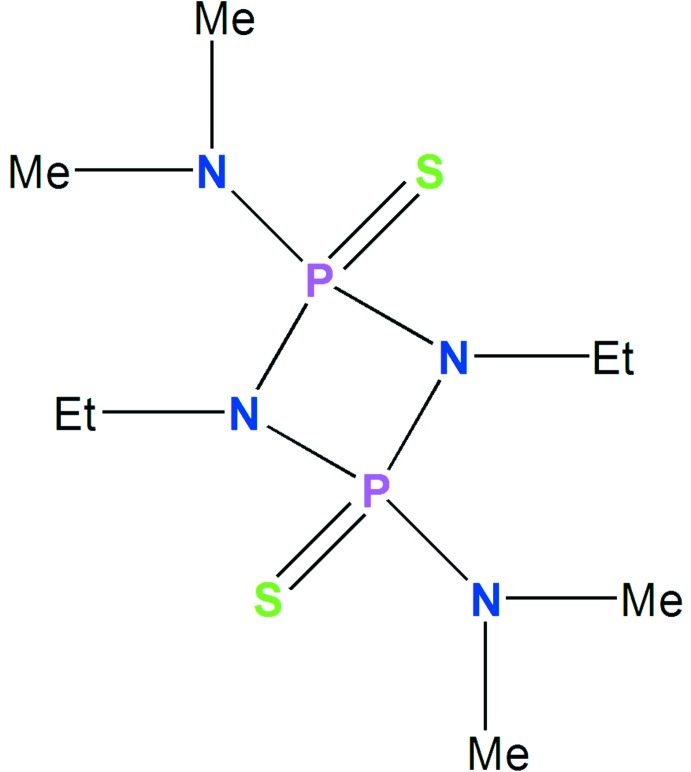


We report here the synthesis, characterization by (^1^H and ^31^P) NMR and energy-dispersive X-ray (EDX) spectroscopies, and a single-crystal structure of a new cyclo­diphosphazane, 1,3-diethyl-2,4-dimethylamine-2,4-di­thiocyclo­diphosphazane, [EtNP(S)NMe_2_]_2_ (I). In order to evaluate the nature of the inter­molecular inter­actions in the crystal packing and their associated energies, detailed analyses of Hirshfeld surfaces (HS) and fingerprint plot (FP) calculations were performed (Spackman & McKinnon, 2002 ▸; Parkin *et al.*, 2007 ▸; Rohl *et al.*, 2008 ▸; Spackman & Jayatilaka, 2009 ▸).

## Structural commentary   

**Figure 1:**
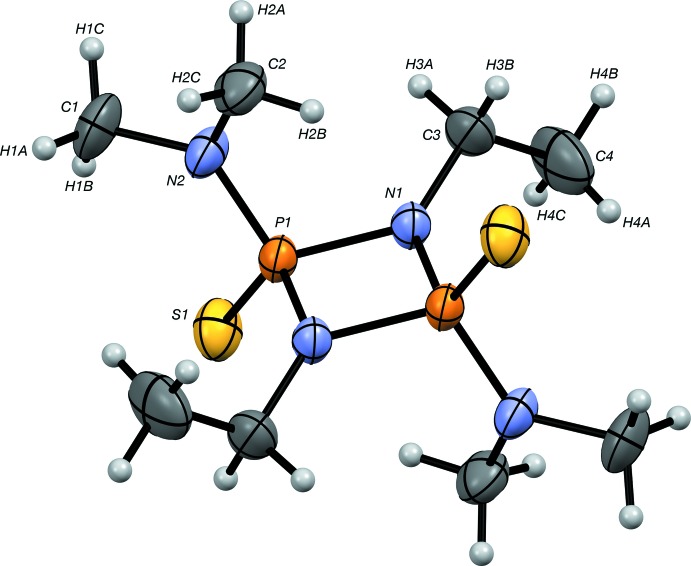
The mol­ecular structure of (I). Atomic displacement parameters for the non-H atoms are drawn at the 30% probability level. Unlabelled atoms are related to labelled ones by the symmetry operation −*x* + 1, −*y* + 1, −*z*.

**Figure 2:**
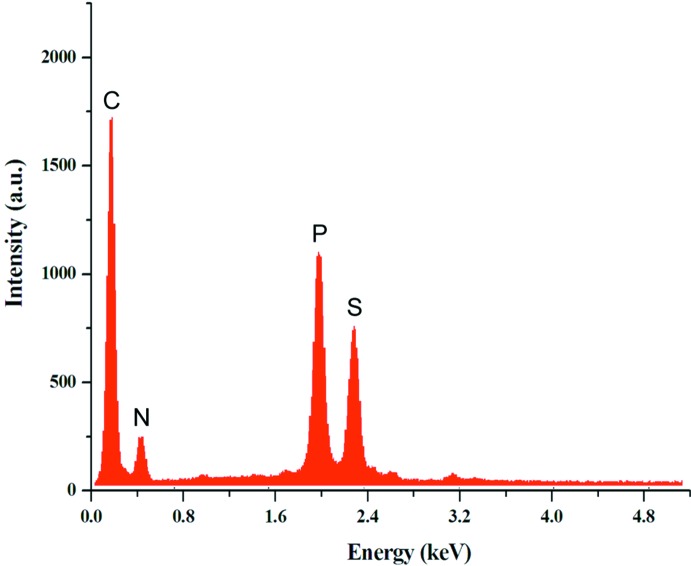
The EDX spectrum of (I), showing the presence of C, N, P, and S.

The mol­ecular structure of (I) is shown in Fig. 1 ▸, selected crystallographic data are presented in Table 1 ▸, and an EDX spectrum confirming the presence of C, N, P and S is shown in Fig. 2 ▸.

Each phospho­rus atom is bonded to one sulfur and three nitro­gen atoms, which are linked to methyl or ethyl groups. Atoms P1 and N1 form a centrosymmetric cyclic P_2_N_2_ arrangement about an inversion center (½, ½, 0). The P1—N1 distances in the ring [1.6856 (17) and 1.6719 (16) Å] are longer than the P1—N2 distance [1.6325 (19) Å], and the P1—S1 distance is 1.9291 (9) Å. These geometric parameters are in agreement with those observed in related non-cyclic and cyclic neutral ligands (Hill *et al.*, 1994 ▸; Alouani *et al.*, 2002 ▸; Peulecke *et al.*, 2009 ▸; Chandrasekaran *et al.* 2011 ▸).

With regard to the conformation of (I), its structure differs from that of P_2_S_2_N_5_C_9_H_27_ (S-NIPA) (Benabicha *et al.* 1986 ▸) primarily by the existence of the P_2_N_2_ ring. The literature also shows several similar ligands, for example *trans-*[(EtNH)P(S)NEt]_2_ (Hill *et al.* 1994 ▸) and *cis*-P_2_S_2_N_4_C_20_H_42_ (Chandrasekaran *et al.* 2011 ▸). The most similar known ligand to (I) is the cyclic mol­ecule *trans-*[(EtNH)P(S)NEt]_2_ (Hill *et al.* 1994 ▸). The two mol­ecules differ in the environments of the nitro­gen atoms, which are all bound to ethyl groups in *trans-*[(EtNH)P(S)NEt]_2_, the peripheral carbons of which are all disordered.

## Supra­molecular features   

**Figure 3:**
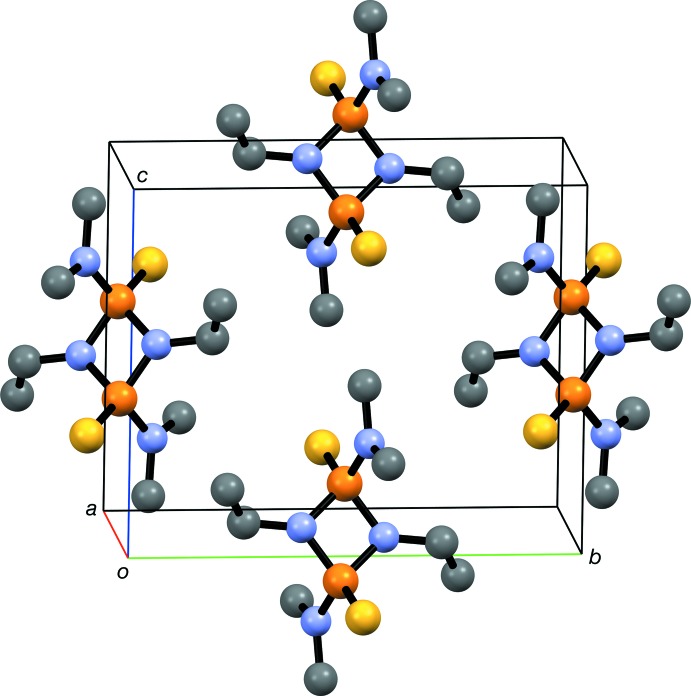
Perspective view of part of the crystal structure of (I), viewed approximately down the *a* axis. H atoms have been omitted for clarity.

A perspective view of (I) is presented in Fig. 3 ▸. Although there are several intra- and inter­molecular close contacts of the form C—H⋯*A* (*A* = S, N), no classical hydrogen bonds are found and the dominant inter­actions are van der Waals contacts.

## Hirshfeld surface analysis   

Organic small mol­ecule crystal packings are often dominated by a particular type of inter­action, *e.g*. hydrogen bonding or van der Waals contacts. However, the overall crystal packing is determined by a combination of many forces, and hence all of the inter­molecular inter­actions of a structure should be taken into account. Visualization and exploration of inter­molecular close contacts of a structure is invaluable, and this can be achieved using the Hirshfeld surface (Spackman & McKinnon, 2002 ▸; Spackman & Jayatilaka, 2009 ▸). A large range of properties can be visualized on the Hirshfeld surface with the program *CrystalExplorer* (Wolff *et al.*, 2012 ▸), including *d*_e_ and *d*_i_, which represent the distances from a point on the HS to the nearest atoms outside (external) and inside (inter­nal) the surface, respectively.

Inter­molecular distance information on the surface can be condensed into a two-dimensional histogram of *d*_e_ and *d*_i_, which is a unique identifier for mol­ecules in a crystal structure, and is known as a fingerprint plot (Parkin *et al.*, 2007 ▸; Rohl *et al.*, 2008 ▸). Instead of plotting *d*_e_ and *d*_i_ on the Hirshfeld surface, contact distances are normalized in *CrystalExplorer* using the van der Waals radius of the appropriate inter­nal (*r*_i_^vdw^) and external (*r*_e_^vdw^) atom of the surface:

*d*_norm_= (*d*_i_ − *r*_i_^vdw^)/*r*_i_^vdw^ + (*d*_e_ − *r*_e_^vdw^)/*r_e_* ^vdw^.

**Figure 4:**
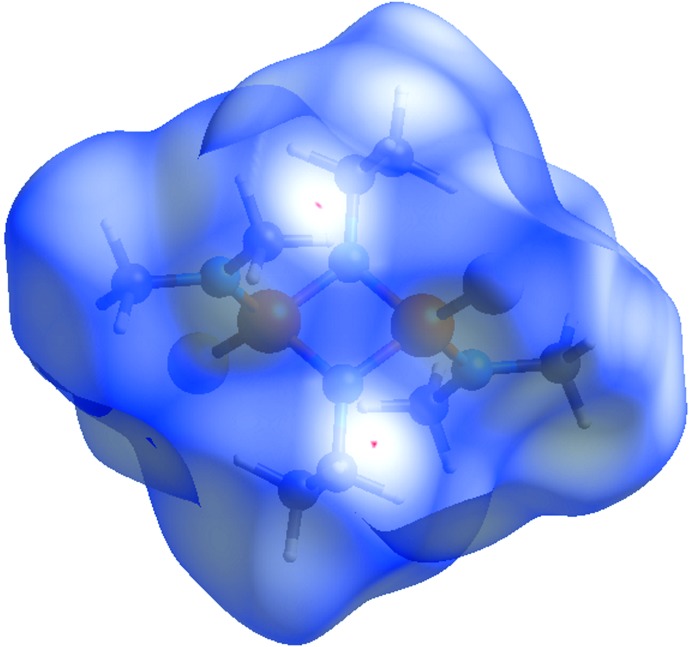
View of the three-dimensional Hirshfeld surface (HS) of (I) mapped with *d*_norm_.

**Figure 5:**
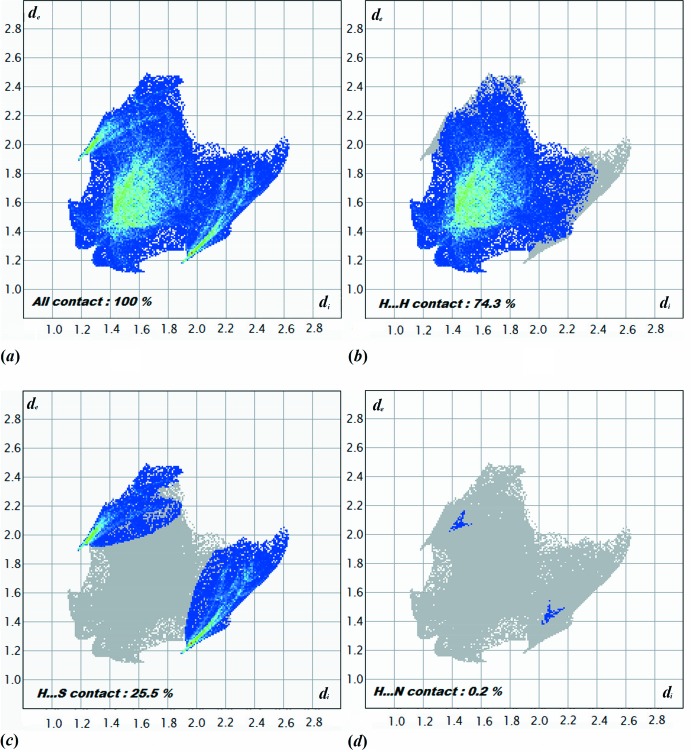
The two-dimensional fingerprint plots of (I), showing (*a*) all inter­actions, and delineated into (*b*) H⋯H, (*c*) H⋯S and (*d*) H⋯N inter­actions [*d*_e_ and *d*_i_ represent the distances from a point on the HS to the nearest atoms outside (external) and inside (inter­nal) the surface, respectively].

For (I), the three-dimensional HS mapped over *d_norm_* is given in Fig. 4 ▸. Contacts with distances equal to the sum of the van der Waals radii are shown in white, and contacts with distances shorter than or longer than the related sum values are shown in red (highlighted contacts) or blue, respectively. Two-dimensional FP plots showing the occurrence of all kinds of inter­molecular contacts are presented in Fig. 5 ▸ *a*.

**Figure 6:**
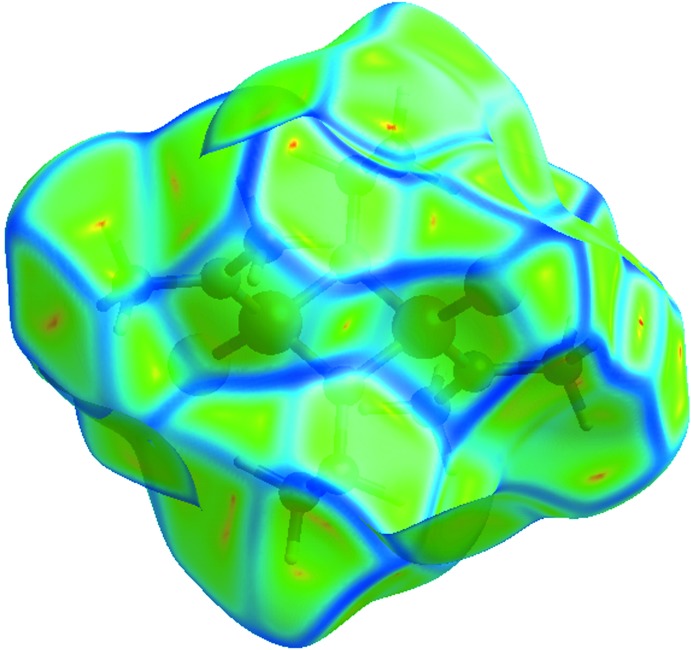
Hirshfeld surface of (I) mapped over curvedness.

The H⋯H inter­actions are shown on the three-dimensional HS as white spots. These contacts appear in the middle of the scattered points in the two-dimensional FP (Fig. 5 ▸ *b*), and represent the most significant contribution to the overall three-dimensional HS (74.3%). Significant H⋯S/S⋯H inter­actions (25.5%) can also be seen, indicated by the pair of wings in the two-dimensional FP with a prominent long spike at *d*_e_ + *d*_i_ ∼ 1.9Å (Fig. 5 ▸ *c*). The H⋯N/N⋯H inter­actions are shown on the three-dimensional HS marked with a blue spot for long contacts. These comprise only 0.2% of the total Hirshfeld surface, and are represented by two symmetrical narrow pointed spikes with *d*_e_ + *d*_i_ ∼ 2 Å (Fig. 5 ▸ *d*). The presence of these inter­actions may also be shown by the Hirshfeld surface mapped as a function of curvedness (Fig. 6 ▸).

## Synthesis and crystallization   

All reagents and solvents were obtained from commercial sources and used without further purification. The synthesis of (I) was carried out in three steps:

• Step 1: Addition of pyridine dropwise to a solution in anhydrous heptane of 2 mol of (EtNH_2_HCl) and 2 mol of PCl_3_ at 268 K, gave precipitation in the form of a salt. Then, the reaction mixture was refluxed for 24 h. An oil was obtained after filtration of the pyridinium salt and evaporation of the heptane and the excess PCl_3_. This step corresponds to the formation of P_2_N_2_ cycle, according to the bibliographic data (Chandrasekaran *et al.* 2011 ▸; Hill *et al.* 1994 ▸). All these operations were conducted under a nitro­gen atmosphere to avoid hydrolysis of the chlorinated compounds. The yield of this step is 85% with respect to ethyl­ammonium chloride.

• Step 2: At a temperature of 263 K, 1 mol of the synthesized [EtNPCl]_2_ was added dropwise to an ether solution containing 2 mol of di­methyl­amine, 2 mol of tri­ethyl­amine and 4-di­methyl­amino­pyridine (4-DMAP) as catalyst. After 10 h of agitation, Et_3_NHCl was precipitated. Filtration of the salt and evaporation of the ether gave an oil. All these operations were conducted under a nitro­gen atmosphere. The yield of this step is 40%.

• Step 3: The sulfurization of [EtNPNMe_2_]_2_ with 2 mol of sulfur gave the final product, 1,3-diethyl-2,4-dimethyl-2,4-di­thio-cyclo­diphosphazane (I), in a yield of about 80%.

Crystallization was carried out from ethanol by slow evaporation at room temperature. After one week, yellow single crystals suitable for X-ray diffraction analysis were obtained. A qualitative EDX analysis on some crystals confirmed the presence of C, N, P and S.

Yield: (80%), yellow solid, ^1^H NMR (300 MHz, CDCl_3_): *δ* (ppm) 1.17 (*t*, 1H, ^3^ *J*_HH_ = 7.26Hz), 2.91 (*d*, 1H, ^3^ *J*_HP_ = 12.45Hz), 3.03 (*m*, 2H); ^31^P NMR (300 MHz, CDCl_3_): *δ* (ppm) 60.13 (1P).

## Refinement   

Crystal data, data collection and structure refinement details are summarized in Table 1 ▸. H atoms attached to CH_3_ and CH_2_ groups were placed geometrically and refined using a riding model: C—H = 0.96 Å for CH_3_ group with *U*_iso_(H) = 1.5*U*_eq_(C) and C—H = 0.97Å for CH_2_ group with *U*_iso_(H) = 1.2*U*_eq_(C).

## Supplementary Material

Crystal structure: contains datablock(s) I. DOI: 10.1107/S2056989017005187/pk2598sup1.cif

Structure factors: contains datablock(s) I. DOI: 10.1107/S2056989017005187/pk2598Isup2.hkl

Supporting information file. DOI: 10.1107/S2056989017005187/pk2598Isup3.cml

CCDC reference: 1015789

Additional supporting information:  crystallographic information; 3D view; checkCIF report

## Figures and Tables

**Table 1:** Experimental details

Crystal data	
Chemical formula	C_8_H_22_N_4_P_2_S_2_
*M*_r_	300.35
Crystal system, space group	Monoclinic, *P*2_1_/*c*
Temperature (K)	293
*a*, *b*, *c* (Å)	7.1975 (10), 11.448 (2), 9.645 (2)
β (°)	96.39 (3)
*V* (Å^3^)	789.8 (2)
*Z*	2
Radiation type	Mo *K*α
μ (mm^−1^)	0.52
Crystal size (mm)	0.40 × 0.40 × 0.30

Data collection	
Diffractometer	Enraf–Nonius CAD-4
Absorption correction	ψ scan (North *et al.*,1968 ▸)
*T*_min_, *T*_max_	0.999, 1.000
No. of measured, independent and observed [*I* > 2σ(*I*)] reflections	3150, 1724, 1463
*R*_int_	0.016
(sin θ/λ)_max_ (Å^−1^)	0.638

Refinement	
*R*[*F* ^2^ > 2σ(*F* ^2^)], *wR*(*F* ^2^), *S*	0.041, 0.127, 1.08
No. of reflections	1724
No. of parameters	76
H-atom treatment	H-atom parameters constrained
Δρ_max_, Δρ_min_ (e Å^−3^)	0.28, −0.27

Computer programs: *CAD-4 EXPRESS* (Duisenberg, 1992 ▸; Macíček & Yordanov, 1992 ▸), *XCAD4* (Harms & Wocadlo, 1995 ▸), *SHELXS97* (Sheldrick, 2008 ▸), *SHELXL2014/7* (Sheldrick, 2015 ▸), *Mercury* (Macrae *et al.*, 2006 ▸), *WinGX* (Farrugia, 2012 ▸) and *publCIF* (Westrip, 2010 ▸).

## supplementary crystallographic information

### Crystal data

**Table:**

C_8_H_22_N_4_P_2_S_2_	*F*(000) = 320
*M**_r_* = 300.35	*D*_x_ = 1.263 Mg m^−^^3^
Monoclinic, *P*2_1_/*c*	Mo *K*α radiation, λ = 0.71073 Å
*a* = 7.1975 (10) Å	Cell parameters from 25 reflections
*b* = 11.448 (2) Å	θ = 10–15°
*c* = 9.645 (2) Å	µ = 0.52 mm^−^^1^
β = 96.39 (3)°	*T* = 293 K
*V* = 789.8 (2) Å^3^	Prism, yellow
*Z* = 2	0.40 × 0.40 × 0.30 mm

### Data collection

**Table:**

Enraf–Nonius CAD-4 diffractometer	1463 reflections with *I* > 2σ(*I*)
Radiation source: fine-focus sealed tube	*R*_int_ = 0.016
Graphite monochromator	θ_max_ = 27.0°, θ_min_ = 2.8°
ω/2θ scans	*h* = −9→4
Absorption correction: ψ scan (North *et al.*,1968)	*k* = −1→14
*T*_min_ = 0.999, *T*_max_ = 1.000	*l* = −12→12
3150 measured reflections	2 standard reflections every 120 reflections
1724 independent reflections	intensity decay: 1%

### Refinement

**Table:**

Refinement on *F*^2^	0 restraints
Least-squares matrix: full	Hydrogen site location: inferred from neighbouring sites
*R*[*F*^2^ > 2σ(*F*^2^)] = 0.041	H-atom parameters constrained
*wR*(*F*^2^) = 0.127	*w* = 1/[σ^2^(*F*_o_^2^) + (0.0779*P*)^2^ + 0.1315*P*] where *P* = (*F*_o_^2^ + 2*F*_c_^2^)/3
*S* = 1.08	(Δ/σ)_max_ < 0.001
1724 reflections	Δρ_max_ = 0.28 e Å^−^^3^
76 parameters	Δρ_min_ = −0.27 e Å^−^^3^

### Special details

**Table:**

Geometry. All esds (except the esd in the dihedral angle between two l.s. planes) are estimated using the full covariance matrix. The cell esds are taken into account individually in the estimation of esds in distances, angles and torsion angles; correlations between esds in cell parameters are only used when they are defined by crystal symmetry. An approximate (isotropic) treatment of cell esds is used for estimating esds involving l.s. planes.

### Fractional atomic coordinates and isotropic or equivalent isotropic displacement parameters (Å^2^)

**Table:**

	*x*	*y*	*z*	*U*_iso_*/*U*_eq_	
P1	0.50835 (7)	0.50319 (4)	0.13083 (5)	0.0515 (2)	
S1	0.33563 (10)	0.44283 (7)	0.25236 (6)	0.0790 (3)	
N1	0.4319 (2)	0.58738 (14)	−0.00532 (16)	0.0535 (4)	
N2	0.6900 (3)	0.56638 (18)	0.21583 (18)	0.0670 (5)	
C1	0.7415 (5)	0.5568 (3)	0.3657 (3)	0.0952 (9)	
H1A	0.8411	0.5013	0.3838	0.143*	
H1B	0.6352	0.5313	0.4096	0.143*	
H1C	0.7821	0.6316	0.4024	0.143*	
C2	0.8350 (4)	0.6172 (3)	0.1411 (3)	0.0814 (7)	
H2A	0.8611	0.6952	0.1740	0.122*	
H2B	0.7932	0.6190	0.0431	0.122*	
H2C	0.9465	0.5708	0.1569	0.122*	
C3	0.3911 (4)	0.7123 (2)	−0.0151 (3)	0.0779 (7)	
H3A	0.4058	0.7454	0.0781	0.093*	
H3B	0.4816	0.7496	−0.0679	0.093*	
C4	0.2046 (5)	0.7387 (3)	−0.0806 (5)	0.1204 (13)	
H4A	0.1842	0.7000	−0.1692	0.181*	
H4B	0.1921	0.8216	−0.0940	0.181*	
H4C	0.1139	0.7121	−0.0218	0.181*	

### Atomic displacement parameters (Å^2^)

**Table:**

	*U*^11^	*U*^22^	*U*^33^	*U*^12^	*U*^13^	*U*^23^
P1	0.0546 (3)	0.0576 (3)	0.0405 (3)	0.0050 (2)	−0.0029 (2)	0.00125 (18)
S1	0.0763 (4)	0.1040 (5)	0.0580 (4)	−0.0017 (3)	0.0128 (3)	0.0143 (3)
N1	0.0607 (9)	0.0518 (8)	0.0455 (8)	0.0091 (7)	−0.0047 (6)	0.0007 (6)
N2	0.0686 (11)	0.0823 (12)	0.0463 (8)	−0.0038 (9)	−0.0108 (8)	−0.0049 (8)
C1	0.099 (2)	0.130 (2)	0.0502 (12)	−0.0017 (18)	−0.0202 (13)	−0.0108 (14)
C2	0.0660 (13)	0.0974 (18)	0.0771 (15)	−0.0159 (13)	−0.0078 (11)	−0.0025 (14)
C3	0.0919 (17)	0.0540 (11)	0.0833 (15)	0.0127 (11)	−0.0097 (13)	−0.0028 (11)
C4	0.091 (2)	0.087 (2)	0.178 (4)	0.0302 (17)	−0.007 (2)	0.038 (2)

### Geometric parameters (Å, º)

**Table:**

P1—N2	1.6325 (19)	C1—H1C	0.9600
P1—N1	1.6719 (16)	C2—H2A	0.9600
P1—N1^i^	1.6856 (17)	C2—H2B	0.9600
P1—S1	1.9291 (9)	C2—H2C	0.9600
P1—P1^i^	2.5143 (10)	C3—C4	1.450 (4)
N1—C3	1.460 (3)	C3—H3A	0.9700
N1—P1^i^	1.6857 (17)	C3—H3B	0.9700
N2—C2	1.455 (3)	C4—H4A	0.9600
N2—C1	1.456 (3)	C4—H4B	0.9600
C1—H1A	0.9600	C4—H4C	0.9600
C1—H1B	0.9600		
			
N2—P1—N1	108.31 (10)	H1B—C1—H1C	109.5
N2—P1—N1^i^	112.28 (10)	N2—C2—H2A	109.5
N1—P1—N1^i^	83.02 (8)	N2—C2—H2B	109.5
N2—P1—S1	112.81 (8)	H2A—C2—H2B	109.5
N1—P1—S1	120.38 (7)	N2—C2—H2C	109.5
N1^i^—P1—S1	116.69 (7)	H2A—C2—H2C	109.5
N2—P1—P1^i^	117.58 (8)	H2B—C2—H2C	109.5
S1—P1—P1^i^	129.60 (4)	C4—C3—N1	113.7 (2)
C3—N1—P1	131.38 (16)	C4—C3—H3A	108.8
C3—N1—P1^i^	128.43 (17)	N1—C3—H3A	108.8
P1—N1—P1^i^	96.98 (8)	C4—C3—H3B	108.8
C2—N2—C1	113.9 (2)	N1—C3—H3B	108.8
C2—N2—P1	120.47 (15)	H3A—C3—H3B	107.7
C1—N2—P1	124.5 (2)	C3—C4—H4A	109.5
N2—C1—H1A	109.5	C3—C4—H4B	109.5
N2—C1—H1B	109.5	H4A—C4—H4B	109.5
H1A—C1—H1B	109.5	C3—C4—H4C	109.5
N2—C1—H1C	109.5	H4A—C4—H4C	109.5
H1A—C1—H1C	109.5	H4B—C4—H4C	109.5

Symmetry code: (i) −*x*+1, −*y*+1, −*z*.
